# Anti-Skin Cancer Activities of *Apostichopus japonicus* Extracts from Low-Temperature Ultrasonification Process

**DOI:** 10.1155/2017/6504890

**Published:** 2017-05-15

**Authors:** Nam Young Kim, Woon Yong Choi, Soo Jin Heo, Do Hyung Kang, Hyeon Yong Lee

**Affiliations:** ^1^Department of Medical Biomaterials Engineering, Kangwon National University, Chuncheon 200-701, Republic of Korea; ^2^Jeju International Marine Science Center for Research & Education, Korea Institute of Ocean Science & Technology (KIOST), Jeju 63349, Republic of Korea; ^3^Department of Food Science and Engineering, Seowon University, Chungju, Republic of Korea

## Abstract

**Objectives:**

This work aimed to enhance anti-skin cancer activities of *Apostichopus japonicus*, spiky sea cucumber, through ultrasonification extraction process at low temperature.

**Methods:**

Dried *Apostichopus japonicus* was extracted with an ultrasonification process at 50°C and 95 kHz for two hours (UE), and anti-skin cancer activities of the extract from the UE were also compared with those from conventional extraction processes using hot water (WE) or 70% ethanol at 80°C (EE) for 12 hours.

**Results:**

The amount of canthaxanthin in the UE was higher than that in the WE or EE, and its cytotoxicity against human keratinocytes was less than the others. The extract from the UE showed 93.5% inhibition against human malignant cell growth, which was also higher than those from both WE and EE. The extract from the UE demonstrated the ability of inhibiting both cancer cell proliferation and metastasis by downregulating the skin tumor-promoting genes such as Bcl-2, STAT3, and MMP-9.

**Conclusions:**

The ultrasonification process was proved to be effective especially in extracting heat-sensitive marine biomass, *A. japonicus* having higher amounts of canthaxanthin and better anti-skin cancer activities, possibly due to less destruction and high elution of bioactive substances under low temperature extraction condition.

## 1. Introduction

*Apostichopus japonicus* is an echinoderm that is divided into three types based on its color: red *Apostichopus japonicus*, blue *Apostichopus japonicus*, and black *Apostichopus japonicus* [1]. *Apostichopus japonicus* is used as food, mainly in Northeast Asia [2], and has adapted to diverse environments such as bedrock, piles of stones, sand, and mudflat distribution areas [3]. *Apostichopus japonicus* is called the “cold ginseng of the sea” and is in the limelight as a health food not only in Korea but also worldwide; it is a food of great industrial value [4, 5]. For example, *Apostichopus japonicus* has been known to be effective for treating diabetes and asthma, as it activates immune functions in the human body. It could also work in promoting hematogenesis in children and pregnant women because *Apostichopus japonicus* is an alkaline food that has no clear analog among animal foods and contains many inorganic components. *A. japonicus* is considered to be able to prevent the aging of the skin and blood vessels and alleviate constipation because its soft nature facilitates bowel movements [1, 5, 6] Antivirus, anticancer, and immune modulation effects of *Apostichopus japonicus* have also been reported, associated with its antioxidant activity [7–9].

Among the useful substances found in *Apostichopus japonicus*, canthaxanthin is a type of carotenoid that is known for its antioxidative effects [10]. In addition, canthaxanthin has been reported to suppress tumors [11]; it was determined to have inhibitory effects on skin cancer caused by increases in the oxidative stress by ultraviolet rays, and attempts have been made to identify the skin cancer inhibitory effects of *Apostichopus japonicus* [12]. The expression levels of B-cell lymphoma 2 (Bcl-2) that inhibits apoptosis in cancer cells, signal transducer and activator of transcription 3 (STAT3) that promotes cancer cell generation and tumor proliferation, and matrix metalloproteinase-9 (MMP-9) that is involved in cancer cell metastasis in skin cells were often measured to confirm the anticancer activities of the extracts from natural resources [13–15].

In general, relatively low temperature extraction process was preferred to process marine biomass since most of marine resources tend to be vulnerable to heat and to prevent the destruction of bioactive substances, that is, canthaxanthin for the case of *Apostichopus japonicus* [16]. However, there must be disadvantages of the low temperature extraction such as low extraction yield and longer process time as well. To overcome these difficulties, ultrasonification process has been employed because it can facilitate the elution with the energy resulting from the cavitation phenomena caused by the vibrations of the ultrasonic waves and the kinetic energy of the reacting particles and maintaining high mixing effects [17]. Therefore, in this work, ultrasonification extraction method was applied to improve the extraction of a useful component, canthaxanthin from *A. japonicus* even at low temperature, which could result in enhancing its anti-skin cancer activities.

## 2. Methods

### 2.1. Sample Preparation and Chemicals

For the hot water extraction (WE), 100 g of dried *Apostichopus japonicus* was placed into 1 L of distilled water and the components were extracted for 24 hours at 100°C. For the 70% ethanol extraction (EE), the same amounts of *Apostichopus japonicus* were extracted for 12 hours at 80°C (EE). For the ultrasonification extraction (UE), 100 g of dried *Apostichopus japonicus* was placed into 1 L of 70% ethanol and extracted for 2 hours with 95 kHz ultrasonic waves and at 50°C. The extracts from each process were filtered using vacuum filters, sufficiently concentrated using rotary evaporators (EYELA N-1000, Tokyo Rikakikai Co., Tokyo, Japan), and freeze-dried for three days in a lyophilizer (PVTFA 10AT, ILSHINBioBase, Dongducheon, Korea) to form a powder that was used in the experiment.

For estimating various efficacy of the extracts, human keratinocytes (HaCaT, ATCC, Rockville, MD, USA) were cultured in DMEM (Dulbecco's Modified Eagle Medium, Gibco, Carlsbad, CA, USA) supplemented with 10% fetal bovine serum (Gibco, Carlsbad, CA, USA) at 37°C, 5% CO_2_. All reagents used were purchased from Sigma-Aldrich (St. Louis, MO, USA).

### 2.2. Estimation of Canthaxanthin Concentration in the Extracts

The concentrations of canthaxanthin in the extracts from each process were measured by high-performance liquid chromatography (HPLC, Agilent 1260 series, Agilent, Santa Clara, CA, USA) with a Jupiter 5 *μ* C18 3000A (250 × 4.6 mm) column. The mobile phase solvent was prepared by mixing (A) acetonitrile and (B) methanol at a mixing ratio of 7 : 3; the mobile phase was used at 100% from 0 minutes to 30 minutes at a 0.8 ml/min of flow velocity. The results were measured at a 470 nm wavelength to compare with the peak areas of the canthaxanthin standard (Sigma-Aldrich, St. Louis, MO, USA).

### 2.3. Measurement of Cell Cytotoxicity in Normal Keratinocyes

The cytotoxicity of the *Apostichopus japonicus* extracts against human keratinocytes (HaCaT) was determined using the 3-(4,5-dimethylthiazol-2-yl)-2,5-diphenyltetrazolium bromide (MTT) assay [18]. HaCaT cells at a concentration of 3.5 × 10^5^ cells/ml were placed into a 96-well plate and cultured for one day. The culture medium was removed one day later, and the cells were treated with the sample and cultured again for one day. The culture medium was removed again one day later, an MTT solution at a concentration of 200 *μ*g/ml was placed into the 96-well plate, and the cells were cultured for three hours at 37°C with all light blocked. The MTT solution used for the treatment was removed three hours later, and the 96-well plate was washed 2∼3 times using PBS. Next, 200 *μ*l of dimethyl sulfoxide (DMSO) was added to each well of the 96-well plate, which was allowed to sit for 30 minutes to allow the reactions to develop, and the optical density was measured using a microplate reader (Thermo Fisher Scientific, Waltham, MA, USA) at 570 nm to identify the cytotoxicity.

### 2.4. Measurement of Inhibiting Malignant Melanoma Cell Growth

The growth of human skin malignant melanocytomas (SK-Mel-2, ATCC) was measured using the SRB method [19]. The cells were sufficiently cultured in a 96-well plate for one day, and different concentrations of the *Apostichopus japonicus* extract were added to the cells. After the cells were cultured for 48 hours, the SRB method was used, and the optical density was measured using a microplate reader (Thermo Fisher Scientific, Waltham, MA, USA) at a wavelength of 520 nm.

### 2.5. Production of Matrix Metalloproteinase (MMP-9) from Human Skin Fibroblasts

Human skin fibroblasts (CCD-986sk, ATCC) were irradiated with UVB (6.3 J/cm^2^) for one day using UV (Coralife, 35 W) filters. After the irradiation, culture medium containing the *Apostichopus japonicus* extract at various concentrations was added to the 96-well plate, and the skin fibroblasts were again cultivated for one day. The concentration of MMP-9 in the culture medium was measured using a Human MMP-9 ELISA kit (RayBiotech, Norcross, GA, USA). Using the prepared *Apostichopus japonicus* extract culture medium, the MMP-9 generation was measured using a quantitative method based on the standard curves specified in the kit [20].

### 2.6. Measurement of Gene Expression Levels of Skin Cancer-Related Proteins

SK-MEL-2 cells were treated with the extracts at various concentrations, and the total RNAs were separated from the cells 12 hours after the treatment using TRIzol® (Invitrogen, Waltham, Massachusetts, USA). Thereafter, the cDNAs were synthesized using 2 *μ*g of RNA, a random primer, and the PrimeScript 1st strand cDNA Synthesis Kit (Takara Bio, Otsu, Japan). The expression levels of the genes related to skin cancer factors such as Bcl-2 (B-cell lymphoma 2), STAT3 (signal transducer and activator of transcription 3), and MMP-9 (matrix metalloproteinase-9) genes were examined using SYBR Green Real-time PCR Master Mix and RT-PCR (AB 7500, Invitrogen, Waltham, Massachusetts, USA) under the following conditions: 30 sec at 94°C, 30 sec at 61°C, and 30 sec at 72°C [21].

### 2.7. Statistical Analysis

The data in all experiments were repeated three times and analyzed by two-way ANOVA using the Statistical Analysis System (SAS Institute Inc., Cary, NC, USA) program. The minimum significant difference was set to *p* < 0.05.

## 3. Results and Discussion

### 3.1. Canthaxanthin Concentration in the Extracts

The estimated contents of canthaxanthin in the extracts of WE, EE, and UE are shown in Table 1. The content of canthaxanthin in the WE hot water extracts was measured as 1.3% (*W*/*W*), which was the lowest and that in the 70% ethanol extract was 2.8%. However, the content of canthaxanthin from the ultrasonification process was 4.4%, ca. 60% higher than that of the EE. These results imply that the low-temperature extraction process associated with ultrasonification could improve the elution of canthaxanthin from *Apostichopus japonicus*, possibly due to the high energy input that can break down the hard cell walls of the sea cucumber. This content was similar to or even better than those obtained from other works [17].

### 3.2. Cell Cytotoxicity of the Extracts from Different Extraction Processes

**Figure 1:**
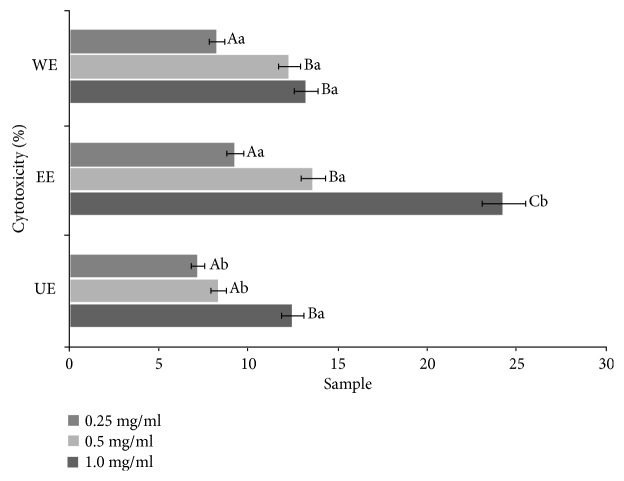
Cytotoxicity of the extracts from three different extraction processes. Mean values ± SD from separate experiments in triplicate are shown. Means with different letters (A–C) within the same concentration are significantly different at *p* < 0.05, and means with different letters (a-b) within same sample are significantly different at *p* < 0.05. WE: water extract, EE: 70% ethanol extract, UE: 70% ethanol extract with ultrasonication process.

The cytotoxicity of the extracts against human keratinocytes, HaCaT cells, is shown in Figure 1. At the maximum concentration of 1.0 mg/ml, the cytotoxicity of the WE and the EE are estimated as 13.26% and 24.30%, respectively. However, the UE showed the lowest cell cytotoxicity of 12.50%, possibly because the low-temperature process could minimize the generation of toxic substances during the extraction process, especially for marine bioresources. Similar patterns have been found in extracting medicinal plants, which are generally more stable in heating processes than marine biomass [22].

### 3.3. Inhibition of Both Melanoma Cell Growth and Production of MMP-9

**Figure 2:**
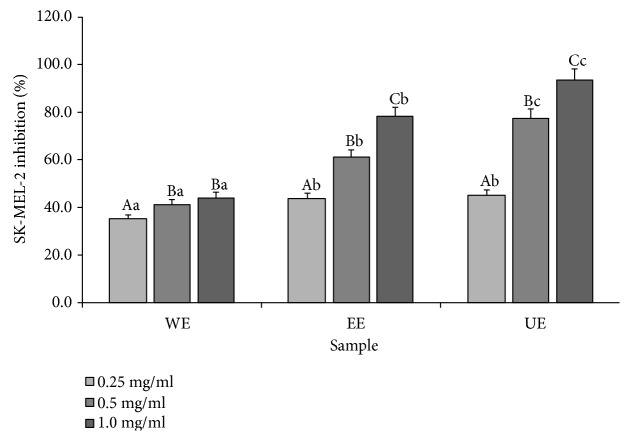
Inhibition of human malignant melanoma cell (SK-MeL-2) growth in treating the extracts from different extraction processes. Mean values ± SD from separate experiments in triplicate are shown. Means with different letters (A–C) within same sample are significantly different at *p* < 0.05, and means with different letters (a–c) within same concentration are significantly different at *p* < 0.05. WE: water extract, EE: 70% ethanol extract, UE: 70% ethanol extract with ultrasonication process.

The effects of the *Apostichopus japonicus* extracts on skin cancer were confirmed, as cell inhibitory effects against the malignant melanocytoma of the skin, SK-Mel-2, were identified using *Apostichopus japonicus* extracts. The results are shown in Figure 2. Hot water extracts showed a maximum inhibition rate of 44.17% at the concentration of 1.0 mg/ml, and the 70% ethanol extracts showed an inhibition rate of 78.30%. The ultrasonicated extracts showed the highest inhibition rate of 95.50%, indicating the improvement of the malignant skin melanocytoma inhibitory effects. In a previous study, where toxicity against SK-Mel-2 cells was identified in a liriope rhizome-derived substance, the inhibition rate was 99.6% at a concentration that was 1000 times the concentration of the *Apostichopus japonicus* extracts in the present study [23]. Based on our resulting concentrations of canthaxanthin, ultrasonification seems to be capable of improving the malignant melanocytoma inhibitory effects and the canthaxanthin concentration of *Apostichopus japonicus* extracts.

**Figure 3:**
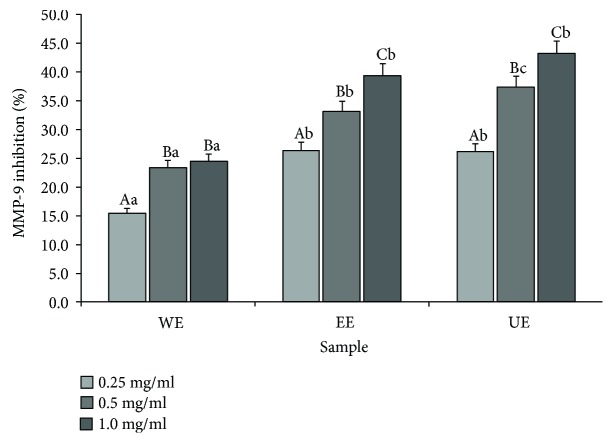
Inhibition of MMP-9 production from human skin fibroblasts with the UVB treatment in adding the extracts from different extraction processes. Mean values ± SD from separate experiments in triplicate are shown. Means with different letters (A–C) within same sample are significantly different at *p* < 0.05, and means with different letters (a–c) within same concentration are significantly different at *p* < 0.05. WE: water extract, EE: 70% ethanol extract, UE: 70% ethanol extract with ultrasonication process.

Experiments were conducted to identify the skin cancer inhibitory effects of *Apostichopus japonicus* extract through its inhibitory effects on MMP-9, which is a gelatinase and is related to cancer cell metastasis [15]; the results are shown in Figure 3. When the inhibition rate of the group treated with only UVB was assumed to be 0% and the inhibition rates of the groups treated with the individual extracts in addition to UVB were measured, it could be observed that the MMP-9 inhibition rates increased with the concentration for all extracts. At the highest concentration, the hot water extracts showed the lowest inhibitory effect, with an inhibition rate of 24.53%, and the 70% ethanol extracts showed better inhibitory effects, with an inhibition rate of 39.48%. However, the ultrasonicated 70% ethanol extracts showed an inhibition rate of 43.27%, a higher inhibition rate compared to the ethanol extracts, indicating that ultrasonification improved the general inhibitory effects of MMP-1.

### 3.4. Downregulation of the Skin Cancer-Related Gene Expression

**Figure 4:**
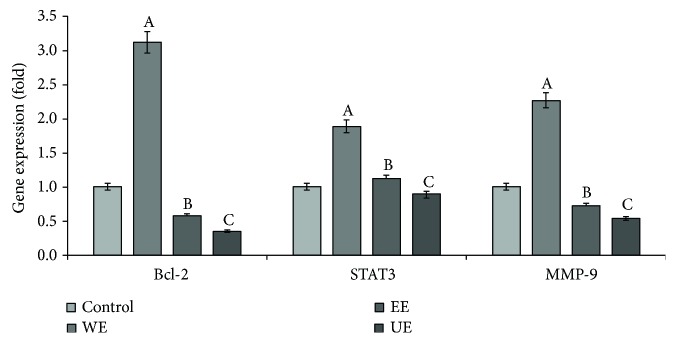
Comparison of the expression levels of skin tumor-promoting genes in treating the extracts from different extraction processes. Mean values ± SD from separate experiments in triplicate are shown. Means with different letters (A–C) within the same gene are significantly different at *p* < 0.05. WE: water extract, EE: 70% ethanol extract, UE: 70% ethanol extract with ultrasonication process.

The skin cancer inhibitory effects of the *Apostichopus japonicus* extracts were evaluated by measuring the expression of Bcl-2 [13], which suppresses apoptosis and leads to cancer cell growth, STAT3 [14], which is involved in the growth and proliferation of tumors, and MMP-9 in skin fibroblasts (Figure 4). When the expression of Bcl-2 in the control, with no treatment, was set to 1.0, the expression in the group treated with the general ethanol extract was 0.58 and that in the group treated with the ultrasonicated extracts was 0.35, indicating that the effects increased with ultrasonification. The expression of STAT3 in the group treated with the general ethanol extracts was 1.12, indicating lower inhibitory effects than the nontreatment groups, but the expression in the group treated with the ultrasonicated extracts was 0.89, indicating inhibitory effects. Finally, both extracts showed effects on MMP-9, with an expression of 0.72 in the group treated with the general ethanol extracts and 0.54 in the group treated with the ultrasonicated extracts. Although the effects of the ethanol extracts and the ultrasonicated ethanol extracts could not be identified, all of the groups treated with the hot water extracts showed higher expression levels than the nontreatment groups, such that no inhibitory effects of the expression of skin cancer genes were identified.

## 4. Conclusion

Marine biomass is generally more sensitive to high temperature than terrestrial plant resources, and it can have a low extraction yield as well generate high levels of toxic substances during the high-temperature extraction processes. However, in this work, it was clearly shown that the low-temperature extraction associated with the ultrasonification process was an efficient process in treating a marine resource, *Apostichopus japonicus*, by showing that the UE extract showed relatively low cell cytotoxicity and also the high elution of an active substance, canthaxanthin, compared to those from two other conventional extraction processes, WE and EE. In addition to the increase of the canthaxanthin concentration, the UE process well demonstrated that the low-temperature extraction decreased the destruction of active components. Similar advantages of ultrasonic extraction have also been reported in other studies [17]. Besides good antitumor effects of the UE extract against human skin cancer cells, downregulation of Bcl-2, STAT3, and MMP-9 genes, which can suppress the induction of skin cancer cell apoptosis and the promotion of cancer cell generation and metastasis, confirmed its effects on preventing the skin cancer. These results could be caused by not only the increase of an active component, canthaxanthin, but also the elution of other useful components through the ultrasonification extraction process even though more detailed studies are necessary. Similar results in other works also showed that ultrasonification extraction process efficiently increased the concentrations of useful components, which can result in improving their biological activities such as anticancer effects, and so forth [24, 25]. It could also tell that these results will be employed to expand the use of marine biomass for other cosmeceutical applications.

## Figures and Tables

**Table 1:** Canthaxanthin contents in the extracts of *Apostichopus japonicus* from various extraction processes.

Sample	Canthaxanthin contents (%)
WE	1.3 ± 0.1^A^
EE	2.8 ± 0.3^B^
UE	4.4 ± 0.6^C^

Mean values ± SD from separate experiments in triplicate are shown. Means with different letters (A–C) are significantly different at *p* < 0.05. WE: water extract; EE: 70% ethanol extract; UE: 70% ethanol extract with ultrasonication process.
